# Comparison of DTL and gold cup skin electrodes for recordings of the multifocal electroretinogram

**DOI:** 10.1007/s10633-022-09912-9

**Published:** 2022-12-20

**Authors:** Theresa Eckermann, Michael B. Hoffmann, Khaldoon O. Al-Nosairy

**Affiliations:** 1 Visual Processing Laboratory, Department of Ophthalmology, Otto-Von-Guericke-University Hospital, Leipziger Str 44, 39120 Magdeburg, Germany; 2 Center for Behavioral Brain Sciences, Magdeburg, Germany

**Keywords:** mfERG, Skin electrode, DTL electrode, P1 amplitude, P1 peak time, SNR

## Abstract

**Objective:**

To compare mfERG recordings with the Dawson–Trick–Litzkow (DTL) and gold cup skin electrode in healthy young and old adults and to test the sensitivity of both electrodes to age-related changes in the responses.

**Methods:**

Twenty participants aged 20–27 years (“young”) and 20 participants aged 60–75 (“old”) with a visual acuity of ≤ 0 logMAR were included. The mfERG responses were recorded simultaneously using DTL and skin electrodes. P1 amplitudes, peak times and signal-to-noise ratios (SNRs) were compared between both electrodes and across age groups, and correlation analyses were performed. The electrode’s performance in discriminating between age groups was assessed via area under curve (AUC) of receiver operating characteristics.

**Results:**

Both electrodes reflected the typical waveform of mfERG recordings. For the skin electrode, however, P1 amplitudes were significantly reduced (*p* < 0.001; reduction by over 70%), P1 peak times were significantly shorter (*p* < 0.001; by approx. 1.5 ms), and SNRs were reduced [(*p* < 0.001; logSNR ± SEM DTL young (old) vs gold cup: 0.79 ± 0.13 (0.71 ± 0.15) vs 0.37 ± 0.15 (0.34 ± 0.13)]. All mfERG components showed strong significant correlations (*R*^2^ ≥ 0.253, *p* < 0.001) between both electrodes for all eccentricities. Both electrodes allowed for the identification of age-related P1 changes, i.e., P1-amplitude reduction and peak-time delay in the older group. There was a trend to higher AUC for the DTL electrode to delineate these differences between age groups, which, however, failed to reach statistical significance.

**Conclusions:**

Both electrode types enable successful mfERG recordings. However, in compliant patients, the use of the DTL electrode appears preferable due to the larger amplitudes, higher signal-to-noise ratio and its better reflection of physiological changes, i.e., age effects. Nevertheless, skin electrodes appear a viable alternative for mfERG recordings in patients in whom the use of corneal electrodes is precluded, e.g., children and disabled patients.

## Introduction

The electroretinogram (ERG) is a decisive tool for the assessment of the functional integrity of the retina in clinical electrophysiology [1]. While conventional electrophysiology, such as the full-field ERG (ffERG), does not allow for a spatially resolved assessment, this can be provided by using multifocal stimulation techniques [2]. Sutter and Tran [3] introduced a multifocal technique of the ERG by utilizing pseudorandom binary m-sequences that enabled spatially resolved functional mapping of the retina, i.e., multifocal ERG (mfERG) [3–9]. The mfERG can be understood primarily as a combination of bipolar cell responses with smaller contributions from cone photoreceptors [10, 11]. It is a well-established clinical technique for the investigation of, e.g., central or paracentral maculopathies, dysfunction induced by hydroxychloroquine, peripheral retinopathies, and local retinal defects [1].

Different corneal electrodes are recommended for mfERG recordings, e.g., fiber, foil, loop and contact lens electrodes [12]. The response characteristics in the mfERG were compared in the previous studies, e.g., Mohidin et al. [13], for different corneal electrodes, but not for skin electrodes. However, the use of skin electrodes has been reported to be more tolerated for ERG recordings in some patient groups, e.g., children and disabled patients [14, 15], than corneal electrodes, e.g., Dawson–Trick–Litzkow (DTL) electrode. Although skin electrodes might therefore provide an alternative to corneal electrodes, there has not yet been a systematic comparison between corneal and skin electrode mfERG-recordings. For other ERG-types, such comparative assessment has been conducted. The previous studies compared the performance of skin to corneal electrodes, e.g., DTL electrodes [16], for conventional electrophysiology, including ffERG [17–22], photopic negative response (PhNR) [23–25], and pattern ERG (PERG) [26]. It was found that both electrodes showed the same waveform characteristics, but amplitude and SNR were smaller for skin electrode recordings [17–24, 26]. Furthermore, unexpected shortening of the peak times was revealed when using skin instead of corneal electrodes [17]. The small stimulus patches usually employed for multifocal stimulation tend to result in comparatively small mfERG responses. This put an additional challenge specifically to the recording of mfERGs with reduced responses due to the choice of the recording electrode. Therefore, further amplitude and SNR reductions induced by the use of skin electrode might render the mfERG inefficient. Furthermore, the mfERG is an important approach with a specific application field in clinical routine diagnostics and hence specific technical requirements, which differ from that of other ERG approaches. Taken together, a dedicated assessment of the dependence of the mfERG responses on the recording electrode is needed. Therefore, the aim of the present study was to compare the response characteristics of the mfERG, i.e., P1 amplitude and peak time, and SNR of skin and corneal electrodes in order to evaluate the utility of skin electrodes as a valid alternative to standard electrodes, i.e., DTL, in clinical practice.

## Methods

### Participants

Forty visually normal participants were included in the study. They were divided into two groups: “young” [20 participants, mean age (range): 22.8 years (20–27)] and “old” [20 participants, 67.25 years (60–75)]. All had best corrected visual acuity (BCVA) of ≤ 0 logMAR measured by EDTRS chart at 4 m. A full eye examination, visual field testing and OCT measurements were performed to exclude the presence of ocular abnormalities, lens opacities and visual field defects. All participants had a refractive error less than ± 4.0 D; only one elder participant was pseudophakic. Participants gave their written consent prior to the study. The procedures followed the tenets of the Declaration of Helsinki, and the study was approved by the ethics committee of the University of Magdeburg, Germany. Only participants with normal visual fields, as assessed employing the Swedish Interactive Threshold Algorithm 24-2 protocol (SITA-Fast) of the Humphrey Field Analyzer 3 (Carl Zeiss Meditec AG, Jena, Germany), and normal retinal structure as assessed via OCT scans (spectral domain OCT with Glaucoma Module Premium edition; Heidelberg Spectralis®, Heidelberg Engineering, Heidelberg, Germany) were included.

### Electrophysiological recordings

#### Procedure and electrodes

Binocular mfERGs were recorded simultaneously with (i) a DTL electrode [16] (DTL Electrode ERG, Unimed electrode Supplies, Ltd, UK) placed across the cornea along the lower lid and (ii) a gold cup skin electrode (10 mm diameter Golden EEG Cup Electrodes, Natus Manufacturing Limited, Ireland) placed on the lower lid 5 mm below the lid margin. Both active electrodes were referenced to a gold-cup skin electrode at the ipsilateral canthus. Gold-cup electrodes were filled with conductive paste (Ten20, WEAVER and Company, USA) and attached to the skin after cleaning with paste (skinPure, NIHON KOHDEN Corporation, Tokyo, Japan) to reduce the resistance of the skin < 5 kOhm. Initially we attempted to also use the RETeval sensor strip (LKC Technologies Inc., Gaithersburg, MD, USA) on the fellow eye as an alternative skin electrode. However, in combination with our amplifier (specified below), mains intrusions at 50 Hz were usually substantial such that the data did not enter analysis in order to keep offline data manipulations to a minimum for the present study.

#### mfERG stimulation

VERIS Science 6.4.9d13 (EDI: Electro-Diagnostic Imaging, Redwood City, CA, USA) was used for stimulus delivery and mfERG recordings. The stimuli were presented on a monochrome monitor (MDG403, Philips; P45 phosphor) driven with a frame rate of 75 Hz. The stimulus pattern comprised 61 hexagons scaled with eccentricity (stretch factor: 12.9) covering central 45° to record mfERGs from 61 separate visual field locations. The hexagons were modulated between white (270 cd/m2) and black (4 cd/m2) according to a pseudorandom m-sequence [3, 6, 7]. The luminance of the gray background was set at 120 cd/m2. All participants were asked to maintain fixation on a central white cross (1.5° diameter). The selected pseudorandom binary m-sequence was 2^15^–1 steps with each step lasting 13.3 ms resulting in a total recording time of 7 min 17 s per recording. The recording process was divided into 32 overlapping segments of 13.65 s to allow the participants to blink and to alleviate steady fixation during the actual recording.

#### mfERG recordings

Recordings followed the ISCEV mfERG standard [12]. For mfERG recording, pupils were dilated with tropicamide 0.5% (Mydriaticum Stulln® UD, Pharma Stulln GmbH, Germany) and phenylephrine hydrochloride 5% (Neo-Synephrine-POS, URSAPHARM Arzneimittel GmbH, Germany) to at least 7 mm diameter. Additionally, a local anesthetic (Conjucain® EDO® 0.4 mg/1 ml, agent: oxybuprocaine hydrochloride 2 mg/ml) was applied for better comfort. Refractive errors were fully corrected for viewing distance of 34 cm. DTL and gold cup electrode were placed as described above. The room light was dimmed, and the recordings were performed binocularly. Electrical signals were amplified by 50 k with a physiological amplifier (Grass Model 12, Astro-Med, Inc., West Warwick, RI, USA), band-pass filtered (low- and high-frequency cutoffs: 3 and 100 Hz) and digitized at 1200 Hz. Any recording segments with breaks of fixation, eye movement or blinks were rejected online and repeated. Two mfERG blocks were recorded for each participant and averaged offline after kernel extraction, as described below, using IGOR 6.21 (WaveMetrics Inc., Lake Oswego, OR, USA).

#### mfERG analysis

For the data analysis, the first-order kernels were extracted using VERIS Science 6.4.9d13. Two iterations of the artefact removal recommended for mfERG analysis with VERIS Science 6.4.9d13 were applied (settings for artifact removal–epoch: 0–225 ms to cover both epochs, that for the signal-magnitude-estimation and that for the noise-magnitude-estimation; included kernels: first-order kernels). Only one eye of each participant was analyzed (20 right eye, 20 left eye). Further analyses were performed using IGOR 6.21 (WaveMetrics Inc., Lake Oswego, OR, USA). The data were digitally filtered (low cutoff: 3 Hz, high cutoff: 100 Hz). Subsequently, traces were averaged across all responses within the same eccentricity bin, i.e., in 5 ring averages from center to periphery, and amplitude and peak time of P1 were determined according to the ISCEV standard [12]. The SNR as defined by Hood and Greenstein [27], which reflects the mfERG magnitude, was calculated as the ratio of the root-mean-square (RMS) of the signal and noise windows (0–75 ms and 150–225 ms, respectively) for all of tested VF locations.

### Statistical analysis

After extraction of the mfERG parameters (P1 amplitude and peak time and SNR), the data were exported from IGOR to SPSS 26 (Statistical Package for the Social Sciences; IBM, Armonk, NY, USA) for further analysis. Three-way repeated measures ANOVAs (RM ANOVA) with the factors ECCENTRICITY, GROUP, and ELECTRODE were applied to the mfERG P1 amplitudes and peak times and the SNR. Because SNRs follow a normal distribution only after logarithmizing [27], they were logarithmized for statistical analysis. Significant effects were specified post hoc via paired *t* tests and corrected for multiple comparisons with the Sidak correction. Correlations between DTL and gold cup electrode measurements were assessed with the Pearson correlation coefficient and variance explained (*r*^2^). To analyze the discriminatory performance of both electrodes between participants of the group of young and old, receiver operating characteristics analyses were conducted to calculate the area under curve (AUC). To check for significant differences, pairwise comparisons of all measures’ AUCs were applied [28].

## Results

### DTL and gold cup electrode: traces overview

**Fig. 1:**
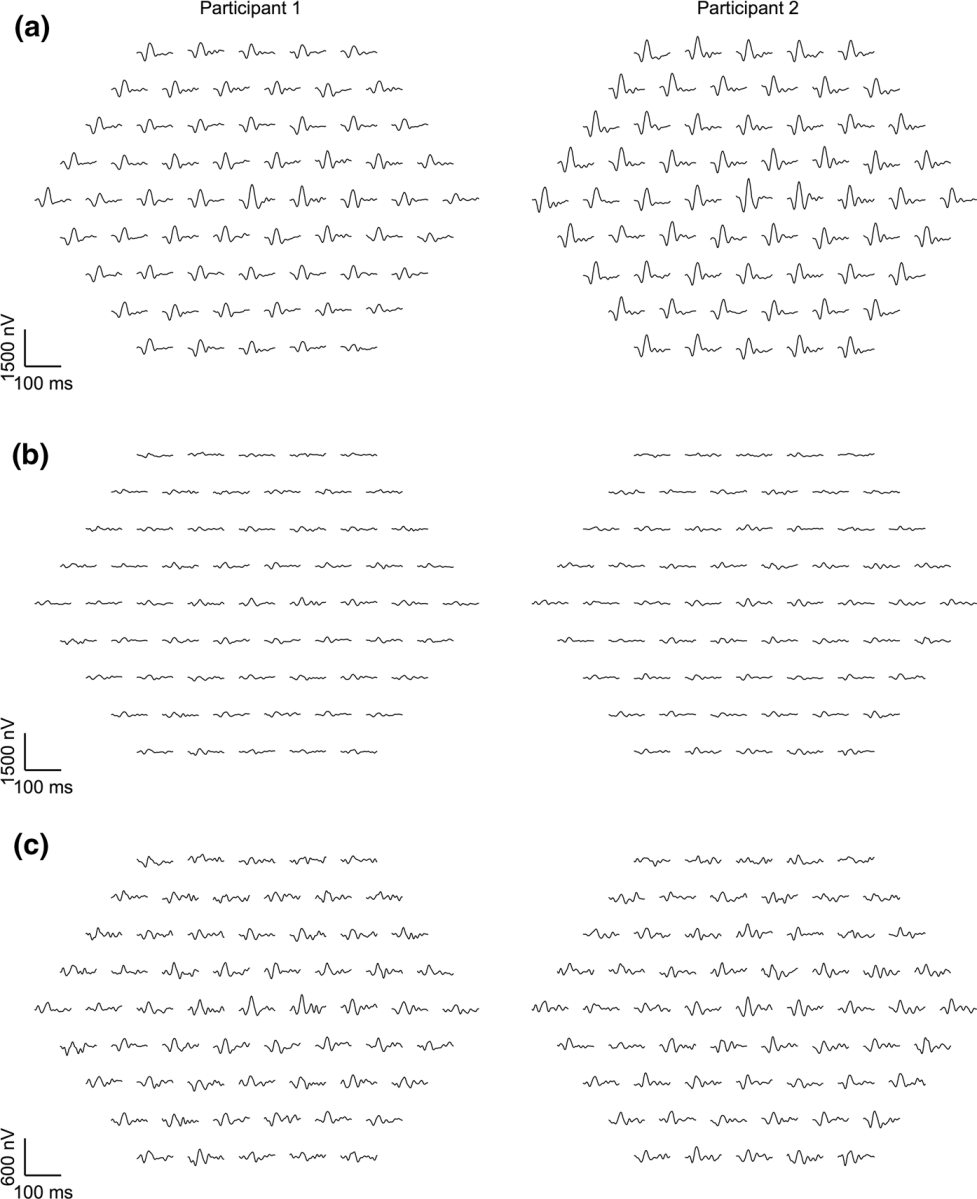
mfERG trace arrays recorded from the left eye of two representative participants with mfERG P1 amplitudes at 25th percentile (participant 1, old) and 75th percentile (participant 2, young). **a** mfERG traces recorded with DTL electrode; **b** mfERG traces recorded with gold cup electrode; **c** mfERG traces recorded with gold cup electrode are displayed with different *y*-axis scaling for a better comparability with the DTL recordings given in (**a**)

**Fig. 2:**
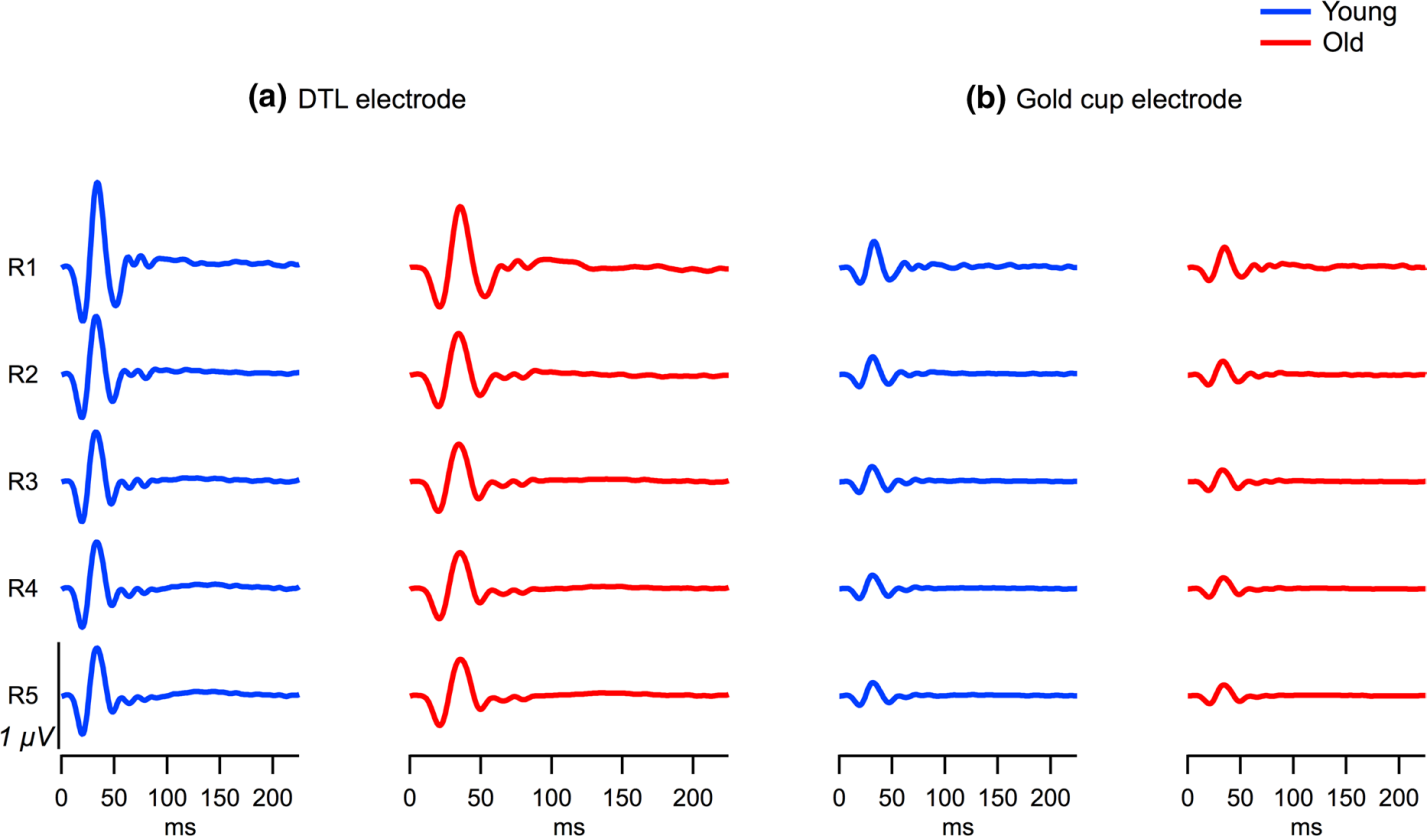
The mfERG grand mean traces across eccentricities for young (blue; *n* = 20) and old (red; *n* = 20) groups recorded with **a** DTL electrode and **b** gold cup electrode. The mfERG responses were reduced for the gold cup compared to DTL electrode in both age groups. With respect to group, for both electrodes, the mfERGs in old were smaller in comparison to young

**Fig. 3:**
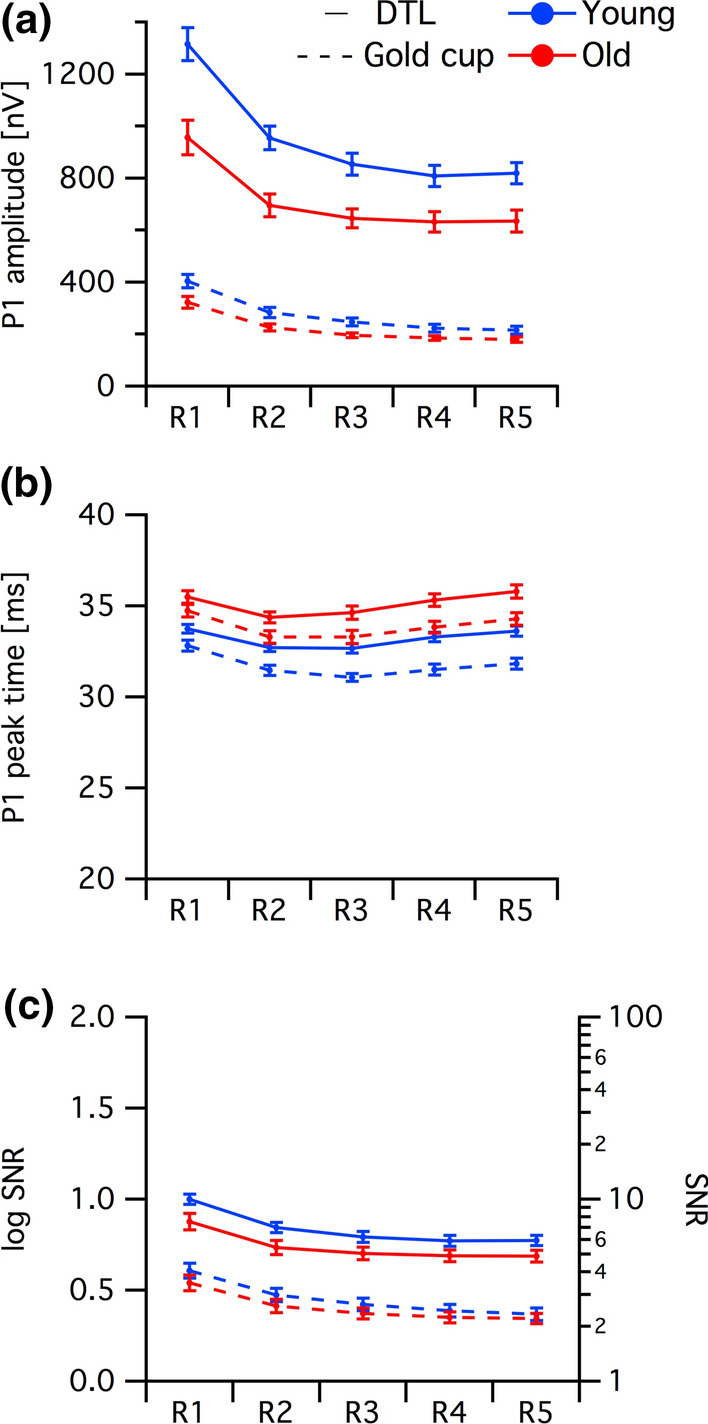
Analysis of mfERG **a** P1 amplitudes, **b** peak times and **c** SNR across different eccentricities depicted in mean ± SEM for old (*n* = 20) and young (*n* = 20). See “Results” for significance levels of the findings. **a** Reduced mfERG P1 amplitudes were evident for all eccentricities for gold cup compared to DTL-electrodes in both groups. Additionally, mfERG amplitudes were reduced for old compared to young in both electrodes. **b** Reduced peak times were evident for all eccentricities for gold cup compared to DTL-electrodes in both groups. In addition, peak times were increased in the old compared to young for both electrodes. **c** Reduced SNRs were evident for gold cup compared to DTL-electrodes. SNRs were higher in young compared to old in both electrodes

For a qualitative assessment, in Fig. 1, a juxtaposition of the mfERG-trace arrays for both electrodes is depicted for two representative participants (P1-amplitude 25th and 75th percentile [old and young participant, respectively]). DTL and gold cup electrode responses reflected the typical features of mfERG recordings, however, as a scaled down and apparently noisier version for the gold-cup electrode. To illustrate this further, the grand mean ring-averaged mfERG traces for electrodes are juxtaposed for both age groups in Fig. 2, which is in support of the message of Fig. 1. In a quantitative analysis, P1-amplitudes, peak times and SNRs were assessed. For the gold-cup electrode, P1 amplitudes were reduced down to 27.6% (young) and 29.5% (old) compared to DTL-electrode responses [DTL young (old): 841.8 ± 182.1 nV (643.2 ± 170.5 nV), gold cup young (old): 232.6 ± 68.7 nV (189.5 ± 42.9 nV), see Fig. 3a]. Furthermore, peak times were shorter in the skin–electrode recorded responses [DTL young (old): 33.2 ± 1.1 ms (35.3 ± 1.6 ms), gold cup young (old): 31.6 ± 1.1 ms (33.8 ± 1.5 ms)]. Likewise, skin electrodes showed lower SNRs than DTL responses [log DTL young (old): 0.79 ± 0.13 (0.71 ± 0.15), log gold cup young (old): 0.37 ± 0.15 (0.34 ± 0.13), see Fig. 3b/c]. The significance of factors’ effects, i.e., electrode, group and eccentricity, is assessed in the subsequent section.

### Quantitative assessment of mfERGs recorded with skin and DTL electrodes

In order to determine the effects of the selected electrode, three-factor RM ANOVAs (factors: ELECTRODE, GROUP and ECCENTRICITY) were conducted for (i) P1 amplitude, (ii) P1 peak time and (iii) SNR retrieved from the ring average mfERG traces as described in methods (Fig. 3).

(i) The RM ANOVA for the P1 amplitude revealed significant main effects of ELECTRODE [F(1,38) = 579.03, *p* < 0.001], GROUP [F(1,38) = 14.78, *p* < 0.001] and ECCENTRICITY [F(1.3,50.9) = 134.61, *p* < 0.001] and a significant interaction of all three factors, i.e., GROUP × ELECTRODE × ECCENTRICITY [F(1.4,53.3) = 6.6, *p* = 0.007]. (ii) The RM ANOVA for P1 peak times revealed significant main effects of ELECTRODE [F(1,38) = 206.04, *p* < 0.001], GROUP [F(1,38) = 29.21, *p* < 0.001] and ECCENTRICITY [F(2.1,78.6) = 33.01, *p* < 0.001]. Only the interaction of ECCENTRICITY × ELECTRODE [F(2.9,111.4) = 9.19, *p* < 0.001] was significant. (iii) The RM ANOVA for the SNRs indicated significant effects of ELECTRODE [F(1,38) = 266.07, *p* < 0.001] and ECCENTRICITY [F(1.4,53.3) = 133.14, *p* < 0.001]. The only significant interaction was ECCENTRICITY × ELECTRODE [F(2.8,105) = 6.15, *p* < 0.001].

### Correlation of mfERG measures between DTL and gold cup electrodes

**Fig. 4:**
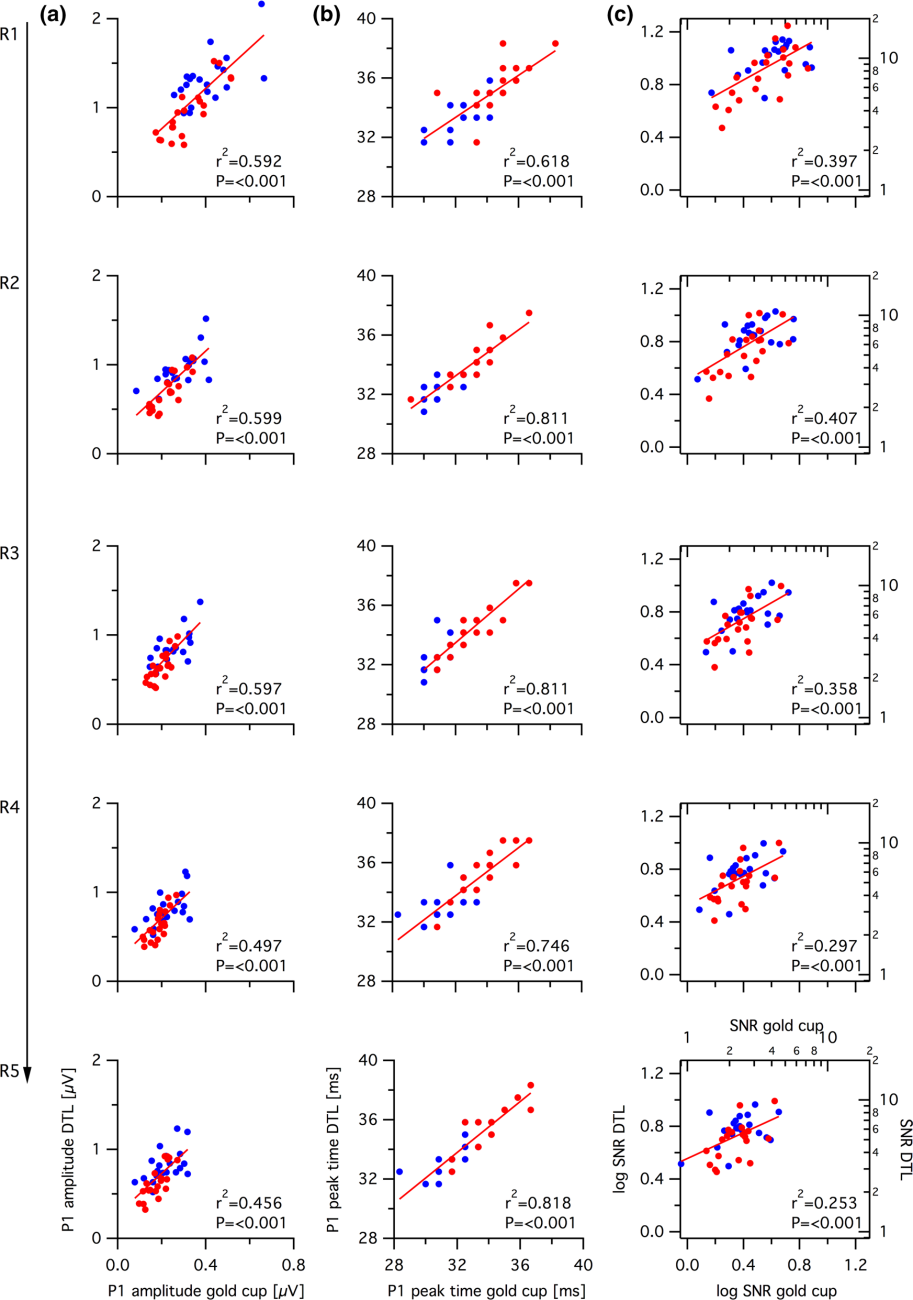
**a** P1 amplitude, **b** P1 peak time, and **c** SNR of DTL electrode vs. gold cup electrode for the young (blue) or old (red) groups (*n*_young_ = 20; *n*_old_ = 20) depicted in an eccentricity dependent manner. Correlation and significance levels are detailed in “Results”

To elucidate the relation between mfERG responses of both electrodes, the correlation between the P1 amplitude (Fig. 4a), P1 peak time (Fig. 4b) and SNR (Fig. 4c) of DTL vs. gold cup electrode was investigated separately for each eccentricity. All mfERG components were strongly and significantly correlated (*R*^2^ ≥ 0.253, *p* < 0.001) between DTL and gold cup electrodes for all eccentricities.

### ROC analysis of mfERG P1 amplitude and peak time between electrodes

**Fig. 5:**
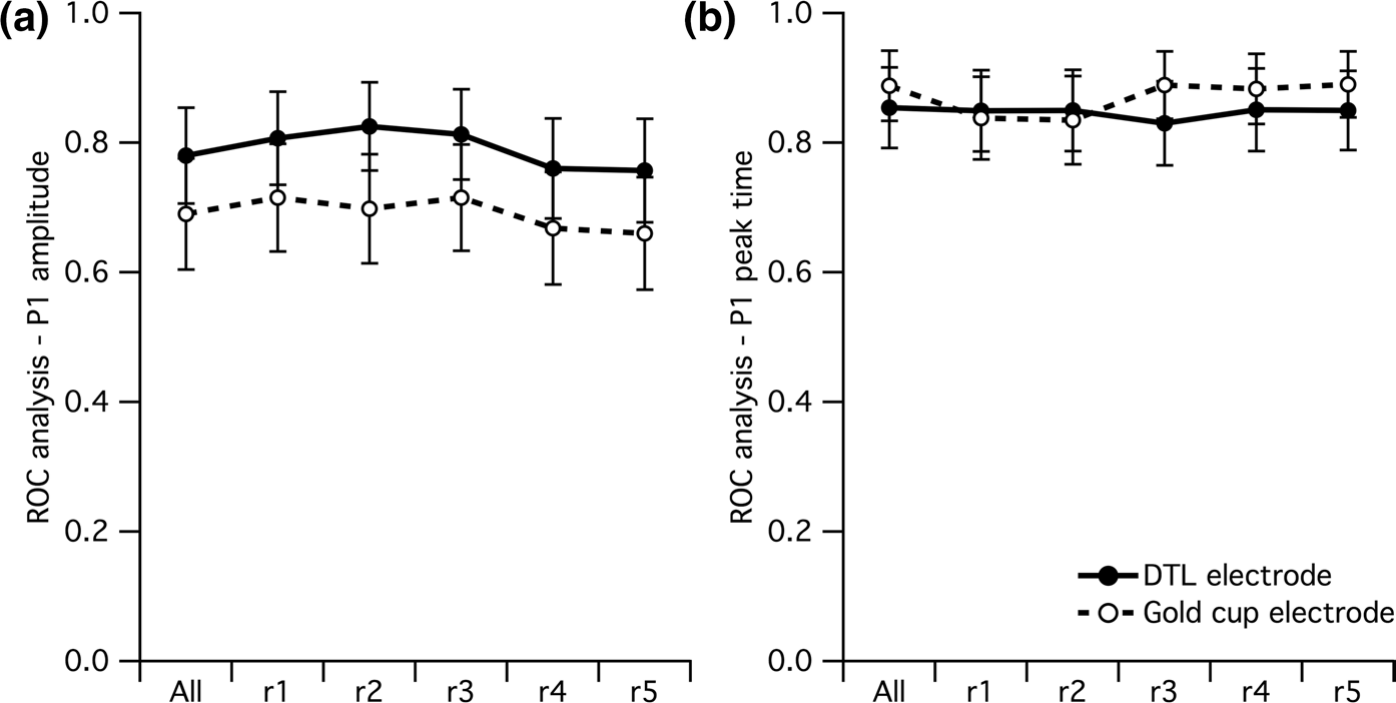
Area under curve for **a** P1 amplitude and **b** peak time (*n*_young_ = 20; *n*_old_ = 20) determined by ROC analysis for both electrodes to test the sensitivity of the electrodes to detect the age-related changes in mfERG. Results are given for **a** P1 amplitudes and **b** peak times across all locations (all) and eccentricities (r1–r5). Significance levels are detailed in “Results”

The discrimination of reduced/delayed responses from normal responses is of critical importance for the application in clinical diagnostics. Using the age dependence as a model to test the discrimination of reduced and delayed responses, we compared the discriminative power of both electrode types between old and young participants. For this purpose, AUC for ROC was calculated to compare the age-related sensitivity of both electrodes in the mfERG parameters, i.e., P1 amplitude and peak time (Table 1, Fig. 5). There was a trend to higher AUC for the DTL electrode in delineating these differences between age groups which, however, failed to reach statistical significance (Table 1).

**Table 1:** AUC of ROC for P1 amplitude and peak time for DTL electrode and gold cup electrode

Electrode	All ± SEM	*R*1 ± SEM	*R*2 ± SEM	*R*3 ± SEM	*R*4 ± SEM	*R*5 ± SEM
P1 amplitude						
DTL	0.78 ± 0.074	0.807 ± 0.072	0.825 ± 0.068	0.813 ± 0.07	0.76 ± 0.077	0.757 ± 0.08
Gold cup	0.69 ± 0.086	0.715 ± 0.083	0.698 ± 0.084	0.715 ± 0.082	0.668 ± 0.087	0.66 ± 0.087
*P*-value	0.219	0.104	0.055	0.15	0.219	0.201
P1 Peak time						
DTL	0.854 ± 0.062	0.849 ± 0.063	0.85 ± 0.063	0.83 ± 0.065	0.851 ± 0.064	0.85 ± 0.061
Gold cup	0.888 ± 0.054	0.838 ± 0.064	0.835 ± 0.068	0.889 ± 0.052	0.883 ± 0.054	0.89 ± 0.051
*P*-value	0.246	0.856	0.573	0.137	0.434	0.236

## Discussion

The responses of the mfERG are influenced by many different factors. Not only diseases of the retina, e.g., maculopathies [4, 5, 29], are for importance, but also other factors like fixation [30–32] and myopia [33]. In this study, we investigated whether the selection of the electrode, i.e., skin vs. corneal, has an influence on mfERG responses. Overall, mfERG-response shapes recorded with DTL and skin electrodes were comparable. However, P1 amplitudes, peak times, and SNR were reduced for the mfERGs recorded with gold cup electrodes. The age-related reduction of the P1 amplitude and increase in P1 peak time in mfERGs is evident for both electrodes.

While there is a lack of studies comparing mfERG parameters between corneal and skin electrodes, the previous studies compared different types of electrodes for other electrophysiological methods, e.g., ffERG or PhNR. In the present study, we demonstrated a 71% reduction in mfERG amplitude for the skin electrode, a finding replicating the previous studies examining other methods. For example, the reduction of skin electrode response reported to be 60% for PhNR [24], 27% for pattern ERG [26], 43–74% for flash ERG [17] and 75% for the ffERG [21], in comparison with DTL.

In line with other studies, we also demonstrated a shortening of peak times, and reduced SNRs when using skin electrodes. Coupland and Janaky [17] observed a shortening of an average of 1.3 ms in the flash ERG when using skin instead of corneal electrodes, which corresponds to the findings of the present study, i.e., 1.56 ms. They suspected that this surprising observation for the peak times of the different electrodes might be related to their spatial distribution with respect to the retinal generator sites. For the comparison of the SNR between the skin and corneal electrodes, other studies obtained results that were comparable to the present study. Tang et al. [23], for example, reported for the skin electrode an SNR-reduction by 38% in the PhNR compared to 60% found in the present study.

The effect of age on the mfERG reported here has also been investigated in other studies. Using DTL-electrodes, Langrova et al. [34] found a decrease in amplitude of 4 to 7.8% per decade depending on the retinal region, which corresponds to the reduction in amplitude we observed between the two age groups that are 4.5 decades apart (reduction in P1 amplitude in old group: DTL [gold cup]: 23.6% [18.15%]). Furthermore, Tam et al. [35] reported a delay in peak time of 0.02–0.03 ms per year, which corresponds to the trend of peak-time prolongation (DTL: 2.09 ms, gold cup: 2.21 ms) we observed in the older group.

Even if the two electrodes differ in some of the relevant mfERG parameters, their comparability can be assessed with a correlation analysis. Here, we demonstrated that both electrodes were closely associated for all mfERG parameters. Likewise, the previous studies have shown that both cornea and skin electrodes deliver reproducible results [22, 36] as long as the position of the respective electrode is kept constant [37, 38].

For clinical diagnostics, it is important that the chosen electrode records a strong signal in the mfERG and is superimposed with noise as little as possible. Skin electrodes are known for smaller amplitudes than DTL electrodes [17–19, 21, 24, 26], because of their location relative to the generators of the recorded signals and their distance from the eye and the lid margin [14, 39]. On the other hand, skin electrodes are less affected by blinking and small eye movements, which is why the responses tend to show smaller standard derivations than those of the cornea electrodes [23]. However, they have higher impedances and are contaminated more easily by muscle potentials of the lid, which leads to a reduced SNR in recordings with skin electrodes, e.g., gold cup electrodes [22]. The reduced SNR represents a major disadvantage of skin electrodes compared to corneal electrodes, e.g., DTL electrode. Even the subsequent use of filters might only help to a limited extent, since strong filtering of data also leads to a loss of information [40–42].

Additionally, in everyday clinical practice, the electrodes must also be able to pick up deviations from normal mfERG responses. In order to compare the discriminative power of the two electrode types, we analyzed the influence of age on the mfERG responses. Age-related changes in the amplitudes seem to have various causes. Both, neuronal factors, e.g., reduction in the photopigment density [34, 43] and optical factors, e.g., changes in the lens (cataract) [35] affect the responses. Peak times were also influenced by the age of the participants according to Langrova et al. [34]. They explained this prolongation in peak time mainly by the slower regeneration of photopigments in older participants. Since not only the amplitudes, but also the peak times are relevant for objective diagnostics of various diseases [4] the discriminative performance of electrodes plays an important role in clinical practice.

Although the performance in discriminating responses across age groups showed no significant difference between the electrodes in our analysis, slightly better results were observed for the DTL electrode. To be able to give a clear recommendation for the use of skin electrodes in clinical diagnostics, further analyses are needed to test this trend in studies with controls versus patients. These should address whether the skin electrode recordings are not only able to detect age-depended variations, but also disease-related mfERG changes.

While analyzing the objective advantages and disadvantages between cornea and skin electrodes, another aspect that needs to be discussed is patient comfort. Even if the use of corneal electrodes is not always preferred, e.g., in children, there were several participants in the present study, who described the skin electrode as uncomfortable and would prefer the DTL electrode over the gold cup electrode for the measurement. The main reasons for this were (i) the preparation of the skin to ensure low impedances which sometimes led to irritation of the skin and (ii) the position on the lower eyelid close to the eye, which was perceived as unpleasant and disturbing. Esakowitz et al. [18] and McCulloch et al. [26] received the same feedback in their studies. To be able to objectively assess and evaluate the comfort of the two electrodes, a systematic evaluation with a questionnaire would be advisable.

In conclusion, this study indicates that both electrode types allow for successful mfERG recordings. The results demonstrated that the use of skin electrodes is an alternative method of recording the mfERG especially in patients in whom the use of a corneal electrode is precluded, e.g., children. Here, skin electrodes, e.g., gold cup electrodes, can simplify mfERG recordings in clinic to objectively assess visual function. However, in compliant patients, the use of the DTL electrode should be preferred due to its larger amplitudes, better SNR and rather better discriminative power.

## Declarations

### Statement on the welfare of animals

This article does not contain any studies with animals performed by any of the authors.
